# First-Principles Study of the Electronic, Vibrational Properties and Anharmonic Effects of Some Si-Based Type-II Binary Clathrates

**DOI:** 10.3390/ma12030536

**Published:** 2019-02-11

**Authors:** Dong Xue, Charley W. Myles

**Affiliations:** Department of Physics and Astronomy, Texas Tech University, Lubbock, TX 79409-1051, USA; charley.myles@gmail.com

**Keywords:** off-center displacement, quartic anharmonicity, self-consistent model

## Abstract

Electronic, vibrational, and anharmonic studies on some binary clathrate A*_x_*Si_136_ (A = Na, K, Rb, Cs; 0 < *x* ≤ 24) are theoretically presented. The Fermi energy lies in the range of 1.1 eV to 1.4 eV for Na*_x_*Si_136_ and increases as stoichiometry (*x*) is tuned from 8 to 12 to 16. The determined isotropic “Mexican-hat” shape of the guest-host potential describing Na motion in the Si_28_ cage indicates the “off-center” position when the temperature is elevated beyond zero. Accordingly, the calculated Na “off-center” displacements correlate well with the X-Ray Diffraction (XRD) data (0.4 Å–0.5 Å) for a similar composition range (0 < *x* < 24). The lack of first-principles analysis on quartic anharmonicity motivates us to initiate a self-consistent model to examine the temperature-dependent rattling frequency *Ω*(*T*) of the guest (Na, Rb). The predicted values of *Ω*(*T*) for Na_24_Si_136_ at 300 K are significantly higher (approximately six times larger) than the value at absolute zero, which contrasts with the case of Rb_8_Si_136_. Moreover, underestimation of the isotropic atomic displacement parameter *U*_iso_ is caused by the temperature-dependent quartic anharmonicity of Na, and this discrepancy might be offset by the square of the “off-center” displacement.

## 1. Introduction

Type-II inorganic clathrates have attracted considerable interest due to their unique crystal structures and promising thermodynamic applications [1,2,3,4,5,6]. These materials have a face-centered cubic (FCC) geometry and an open framework lattice with 136 atoms in the unit cell. Furthermore, the framework atoms in this structure are all in sp^3^ bonding configurations, which form 20- and 28-atom polyhedron “cage” voids that are connected periodically in a 2:1 ratio. Each cage can trap an impurity (“guest”) atom, which is usually from column I or column II of the periodic table. Several type-II clathrates have been synthesized with Si, Ge, or Sn on the framework lattice. The encapsulated guests are weakly bound to the host lattice, which means that their vibrational modes are expected to occur at very low frequencies. Several different type-II Si-based binary clathrates have been synthesized, including Na*_x_*Si_136_ (*x* = 2.9, 5.1, 8.2, 14.7), K*_x_*Si_136_ (*x* = 7.5, 17.8), and Na_16_(Rb,Cs)_8_Si_136_, and many of their properties have been measured [7,8,9,10,11]. One of the interesting results that has been obtained is the fact that (Na,K)_16_Rb_8_Si_136_ [12] shows intermetallic behavior. This is caused by the charge transfer from alkaline metal atom guests to the framework conduction band states, which pushes the Fermi energy up into the lower portion of the conduction bands.

In this paper, we first report the results of a systematic, first-principles computational and theoretical study of the electronic properties of some of the binary type-II Si-based clathrates A*_x_*Si_136_ (*x* = Na, K, Rb, Cs; 0 < *x* ≤ 24). Among the properties, we study the dependence of the pseudogap and Fermi level of the filled intermetallic clathrate Na*_x_*Si_136_ (8 ≤ *x* ≤ 16) on the guest content (*x*). We also study the electronic density of states, which indicates the occurrence of temperature-dependent Knight shift behavior, and when possible, we compare our calculations with some experimental results for (Na,K)_16_Rb_8_Si_136_, which share similar characteristics [13].

One interesting and useful property of the type-II clathrate materials is that their novel cage-structured framework lattice is capable of housing impurity atoms that can form host-guest complexes. Of particular interest are the vibrational properties of these host-guest complexes, specifically the existence of low-frequency guest-associated “rattling” modes. Such modes may help to enhance the thermoelectric (TE) performance by minimizing the phonon contribution to the thermal conductivity. In this paper, we mainly report the results of the vibrational properties of several Si-based type-II clathrate materials. We especially focus on the vibrational modes and their anharmonic effects of various guests in the lattice cages.

If the guest atoms in large cages (28-atom cages) of type-II clathrates are small in comparison to the cage size and if their mass is also relatively small, it is possible that the guest-associated vibrational modes can exhibit significant anharmonic vibrations. To our knowledge, there has been very little attention paid to understanding this anharmonic behavior and its associated “off-center” guest behavior. In this work, we also report the results of a systematic, first-principles study of such anharmonic vibrational modes due to alkali atoms in a large Si_28_ cage in the clathrates A_8_Si_136_ (A = Na, K, Rb, Cs) and Na_24_Si_136_. One goal of this study is to explore the composition (*x*)-dependence of such a dynamically unstable, anharmonic guest-host potential. We find that the effective potential for this situation can be described with a large anharmonicity (large negative quartic coefficient) when the rattling impurity atom is changed from Cs (Rb, K) to Na. We also note that some previous reports of guest anharmonicity did not include a quartic anharmonicity interaction when examining the low-frequency rattling mode of the guests [14,15].

## 2. Computational Approach

Our calculated results are obtained by applying a computational quantum mechanical modeling method, which employs the local density approximation (LDA) to density functional theory (DFT). For such first-principles calculations, we focus on using the Vienna *ab initio* Simulation Package (VASP) [16,17,18,19,20,21], which exploits the Ceperley-Alder exchange-correlation potential, as well as pseudopotentials obtained using the projector augmented wave (PAW) method. The energy cutoff parameter when computing dispersion relations is set to 150 eV for silicon-based materials. This method has been extensively and successively tested in reported calculations. Specifically, K. Biswas et al. previously performed VASP determination of electronic structures regarding Na_16_Rb_8_Si_136_ [12]. In their work, the calculated lattice parameter agrees well with the experimental result [13], while the calculated electronic density of states possessing a sharply peaked feature in the vicinity of the Fermi energy level can be qualitatively linked to the temperature-dependent Knight shift observed for the NMR-active nuclei in Na_16_Rb_8_Si_136_. Furthermore, K. Biswas et al. identified the low-frequency guest “rattling” modes from VASP-computed phonon dispersion relations when studying vibrational properties of Na_16_Rb_8_Si_136_ [22], among which the estimated isotropic mean-square displacement amplitudes (*U*_iso_) relating to Rb and Na are in good agreement with the experiment [11]. Hence, motivated by these reported works, we continued to follow the usage of the LDA to DFT method in the context of VASP while performing *ab initio* evaluations on electronic, vibrational, and anharmonic features of binary system A*_x_*Si_136_ (A = Alkaline metal; 0 < *x* ≤ 24). For our study, a single crystallographic unit cell which contains 34 Si atoms is selected rather than a large clathrate unit cell structure involving 136 framework atoms.

The beginning step of our first-principles calculation starts with structural optimization. This optimization process is achieved by means of a conjugate gradient (CG) method, which relaxes the internal coordinates of the atoms confined in a fixed volume of the FCC unit cell. In the regime of type-II binary clathrate compounds described by cubic space group symmetry (Pm3d), the encapsulated guest atoms are allowed to move freely from their original point positioned by the cage center. It is worth mentioning that such a process for the relaxation and determination of the optimized structure must be repeated many times to achieve a global total minimum energy. Next, we fit limited pairs of LDA-calculated potential energy vs. volume (*E*, *V*) curves to the Birch-Murnaghan equation of state (EOS) to determine the equilibrium energy *E*_0_ and the equilibrium volume *V*_0_. Accordingly, we initiate Brillouin zone (BZ) integration with the help of a 4 × 4 × 4 Monkhorst-Pack *k*-point grid [23] for the purpose of performing the relaxation and ultimately characterizing the equilibrium geometry. The total energy convergence criterion is adjusted to 10^−7^ eV. As soon as the lattice parameter for a material of interest has been determined, the VASP code can readily perform further calculations, pertaining to the Fermi level position, the electronic band structure, and the associated electronic density of states (EDOS).

In the following, we apply our approach to analyze the vibrational features of A_8_Si_136_ (A = Na, K, Rb, Cs), as well as Na_24_Si_136_. The typical technique for obtaining the rattling modes of the above binary clathrates consists of two steps. First, each atom in the single crystallographic unit cell is moved by a small finite displacement (*U*_0_ = 0.02 Å), subject to determination by the dynamical matrix ***D***(***q***). There are two reasons for designating the dynamical matrix requirement. One is based on the fact that ***D***(***q***) is constructed at the gamma (Γ) point [***q*** = (0,0,0)]. On the other hand, ***D***(***q***) can still be calculated for nonzero ***q*** if it is assumed that the matrix elements of ***D***(***q***) vanish for atoms separated by a distance greater than the third nearest neighbor distance [24]. In contrast to the previously mentioned BZ integration method, a new 2 × 2 × 2 *k*-point grid utilizing the *k*-points along certain high-symmetry directions is adopted to compute ***D***(***q***). Second, it is clear that the diagonalization of ***D***(***q***) allows us to find the vibrational eigenvalues (squared frequencies) and eigenvectors.

## 3. Results and Discussion

### 3.1. Electronic Properties

**Figure 1:**
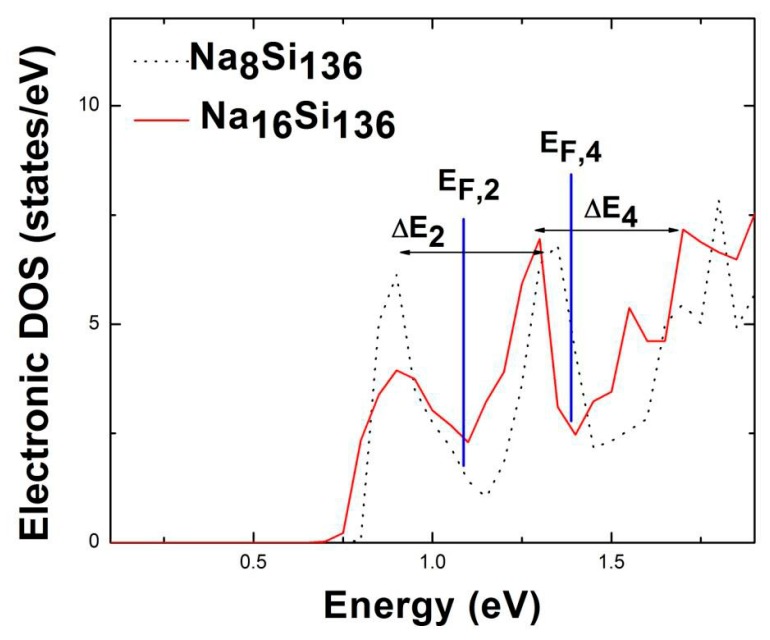
Illustration of the electronic density of states in the lower portion of the conduction band for clathrates Na_8_Si_136_ and Na_16_Si_136._ The Fermi energy levels (*E*_F,2_, *E*_F,4_) are marked by blue lines and quasi-activation energies Δ*E*_2_, Δ*E*_4_ are given regarding sharply peaked structures.

For the purpose of revealing how guest-framework interactions affect the electronic and vibrational properties of clathrate lattices, we initiate our beginning step to provide an EDOS study for the optimized geometries. A previous comprehensive description of the electronic structure associated with Na_16_Rb_8_Si_136_ and K_16_Rb_8_Si_136_ is presented in [12], where K. Biswas et al. calculate the electronic density of states, which displays two sharp peaks and a dip near the Fermi level. Such neighboring peaks have a separation (Δ*E*) at an energy scale of several *k*_B_*T*, which is indicative of the temperature-dependent Knight shift effect observed experimentally for the NMR-active nuclei in Na_16_Rb_8_Si_136_ [25]. Therefore, motivated by this model describing the two-peak form of the EDOS in the vicinity of the Fermi level, we perform a first-principles analysis of the EDOS of Na*_x_*Si_136_ (*x* = 8, 16), which shows an analogous quasi-activation energy Δ*E* when taking the *T*-dependent Knight shift into consideration. Figure 1 illustrates the electronic density of states in the lower portion of the conduction band for Na_8_Si_136_ and Na_16_Si_136_, where the corresponding Fermi energies are denoted by *E*_F,2_ and *E*_F,4_. Here, quasi-activation energy Δ*E*_2,4_ is predicted to lie in the range of approximately 0.4 eV to 0.46 eV. Moreover, the predicted Δ*E*_2_ of Na_8_Si_136_ and Δ*E*_4_ of Na_16_Si_136_ are similar to that of K_16_Rb_8_Si_136_ (Δ*E* ~ 0.41 eV) from a previous work [12].

**Figure 2:**
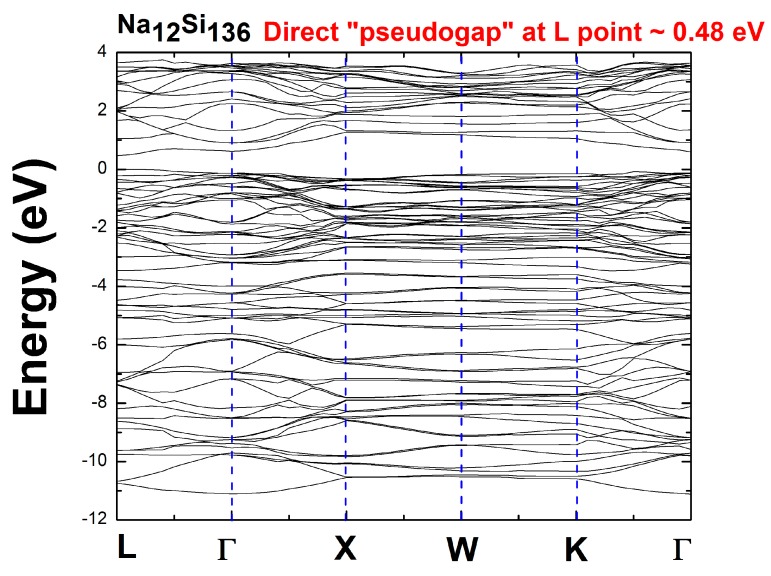
Electronic band structure of Na_12_Si_136_.

In view of the filled intermetallic clathrate Na_12_Si_136_, we found the existence of a direct “pseudogap,” which is defined as the energy difference between the top of the valence band and the bottom of the conduction band at the same L point from the band structure (BS) perspective. Specifically, our DFT computation shows that the pseudogap is approximately equal to 0.48 eV. Figure 2 shows the calculated BS spanning over various Brillouin zone points, illustrating the direct pseudogap behavior.

**Figure 3:**
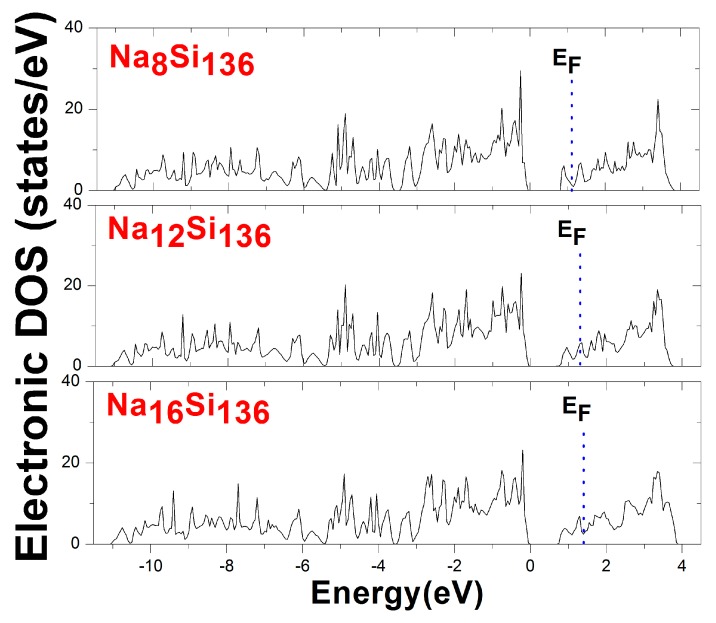
Electronic density of states (DOS) of the clathrate system Na*_x_*Si_136_, where *x* = 8, 12, 16. The Fermi energy levels (*E*_F_) are denoted by dotted lines.

One of the electronic characteristics of the Na*_x_*Si_136_ system is understood through the variation of the Fermi energy level, whose position is shifted into a deep portion of the conduction band as *x* ranges from 8 to 12 and 16 in Figure 3. Furthermore, the shapes of the predicted EDOS profiles for these three filled clathrates are roughly identical and remain nearly independent of the guest composition *x*. In particular, the energy states occupying the valence band in three materials seem to be populated in a significantly consistent manner.

### 3.2. Vibrational Properties and Anharmonic Effects

Calculations of the “rattling” vibrational modes of the alkali metal atom guests in A*_x_*Si_136_ (A = Na, K, Rb, Cs; 0 < *x* < 24) can reveal very interesting basic physics, such as lattice dynamics and guest-host coupling. The main idea of the “rattler” concept originates from the fact that loosely bound guest atoms encapsulated in the “oversized” (28-atom) cages in the type-II clathrates vibrate and produce “localized” modes that are capable of efficiently scattering heat-carrying acoustic phonons [26,27]. Thus, the rattling behavior of the alkali metal atom guests can potentially participate in reducing the material thermal conductivity to a glass-like level, as suggested by Slack’s “Phonon Glass Electron Crystal” (PGEC) criteria [28].

Our study of the vibrational properties of the filled clathrate A*_x_*Si_136_ is divided into two parts, as follows. First, the VASP code is used to calculate the phonon dispersion curves, along with the effective potential energy diagrams, which may contain apparent anharmonicity at zero temperature, depending on the guest (A) type. Second, the significant anharmonicity of the motion of the Na rattling guests in the Si_28_ cages in Na*_x_*Si_136_ is investigated in detail with the aid of a self-consistent phonon model to reveal its temperature dependence. The results predict that these anharmonic effects can strongly affect the force constants for the guest vibrational modes.

The LDA-calculated result correlating to the guest vibrational mode from the phonon dispersion relations in Na_4_Si_136_ agrees fairly well with the data obtained from inelastic neutron scattering (INS) experiments [29]. Specifically, the low-lying vibrational (“rattling”) frequencies of the Na guests occur in a narrow band centered at approximately 50 cm^−1^, which are approximately 4% lower than that of the experimentally observed mode (52 cm^−1^). In addition, an effective force constant *K* for the rattling frequency *ω* in the harmonic approximation (HA) may be obtained by assuming *ω* = (*K*/*M*)^1/2^, where *M* is the atomic mass of the guest. For Na_4_Si_136_, this gives *K* = 0.44 eV/Å^2^ from the first-principles viewpoint for the Na vibrations in the Si_28_ cages.

To gain insight into the anharmonic effects associated with the Na guest vibrations in the Si_28_ cages, we carried out a method that can be summarized as follows. Using the LDA to generate the effective guest-host potential energy for an Na guest in an Si_28_ cage acts as the first step. Then, to approximately treat the lowest order anharmonic effects present in the guest-host interaction, we expand this effective potential in a power series in the guest atom displacements, keeping only the second and fourth order terms. That is, we assume that the guest-host potential energy *V*_eff_ can be approximately written as *V*_eff_ = ½ *K*_iso_*r*^2^ + ¼ *Ɛ*_iso_*r*^4^, where *r* is the displacement from equilibrium and *K*_iso_ and *Ɛ*_iso_ represents empirical constants determined by fitting to the effective potential energy. Only even order terms are kept in this polynomial expansion because of the symmetry of the energy function.

**Figure 4:**
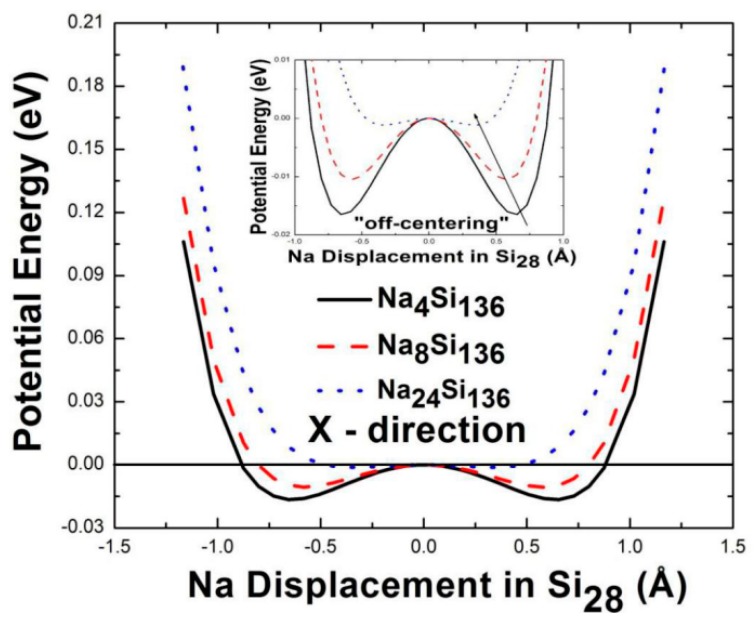
The calculated isotropic Mexican-hat shaped potential-energy curves for Na vibrations in the large 28-atom cages in the clathrate system Na*_x_*Si_136_ (*x* = 4, 8, 24). X represents the motion direction with respect to the Na atom.

In this paper, the procedure used to determine the guest-host framework effective potential energy curves displaces one Na guest atom inside an Si_28_ cage while keeping all remaining atoms in the unit cell fixed. More concretely, each Na is displaced a distance from the center of the Si_28_ cage. In all cases, this displacement distance is strictly restricted to the range of 0 to Δ*r*, where the upper limit Δ*r* describes the “excess” radius of that cage. Our previous work [15,26] showed that the “excess” radius of the Si_28_ cage is Δ*r* = 1.83 Å for Na vibrations. Therefore, our calculation is performed in the Na*_x_*Si_136_ system for Na guests moving in the X direction, but limited to the range of 0 to approximately 1.2 Å and repeated for guest compositions *x* = 4, 8, and 24. In the coordinate system considered here, the X and Y axes are chosen to be aligned in a plane that is parallel to the plane of the hexagon, while the Z axis is perpendicular to the hexagon in the 28-atom cage. The resulting effective potential energy curves for the compositions *x* = 4, 8, and 24 are shown in Figure 4. The results for motion along the X direction are shown. By symmetry, the effective potential energies for the Y-directed and Z-directed motions are nearly identical to that for X-directed motion, indicating the isotropic behavior of the guest vibration.

Here, for Na_4_Si_136_ and Na_8_Si_136_, the results of this procedure predict that the effective potential energy has an obvious “bump” at the cage center (zero Na displacement in Si_28_). Additionally, each of these energy curves in Na*_x_*Si_136_ (*x* = 4, 8) has a “Mexican-hat” (double valley) shape. This form of the potential energy shape shows that Na guests in the Si_28_ cages are dynamically unstable at the cage center. Thus, these calculations predict that, at elevated temperatures, thermal excitation can induce Na guests to move off the cage center to a site with a lower potential energy. In other words, these calculations find that the “localized” off-center minimum valley of the effective potential curve is approximately 0.017 eV and 0.01 eV below zero for Na_4_Si_136_ and Na_8_Si_136_, respectively. That is, the effective potential energy valley depth is predicted to be approximately 0.66 *k*_B_*T* and 0.39 *k*_B_*T* at room temperature for Na_4_Si_136_ and Na_8_Si_136_, respectively. It is likely that the minimum energy valley causes the “off-center” position of Na guest to form due to increased temperature. This kind of anharmonic effect might be correlated with the measured strong temperature dependence of the isotropic atomic displacement parameter *U*_iso_, as reported in References [5,30].

This predicted “off-center” position for Na in the Si_28_ cages in Na_x_Si_136_ can, in principle, be correlated with the experimentally measured guest equilibrium location at various temperatures. However, the disorder of the guest position makes the understanding of such “off-center” sites complex. Similar effects have been observed in some type-I clathrates, including Sr_8_Ga_16_Ge_30_, Ba_8_Ga_16_Ge_30_, and Eu_8_Ga_16_Ge_30_ [5,11,13,15,25,27,31]. The arrow in the inset of Figure 4 indicates that the “localized” off-center minimum valley is shifted towards the cage center when the Na content is changed from 4 to 8 to 24, despite the fact that apparently weakened double-valley behavior exists in the effective potential energy curve of Na_24_Si_136_ compared to others.

**Figure 5:**
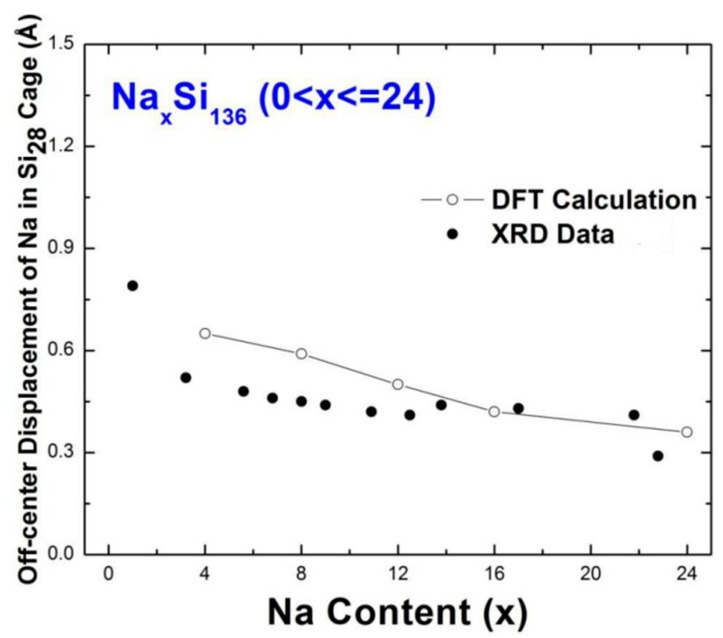
DFT-calculated “off-center” displacements (open circles) of Na in the Si_28_ cages in Na*_x_*Si_136_ as a function of Na composition (*x* = 4, 8, 12, 16, 24), along with XRD measured displacements (solid dots) by Beekman et al. [31].

Considerable experimental work on Na*_x_*Si_136_ by Beekman and collaborators focused on detecting the isotropic, “off-center” displacement of Na in Si_136_ and studying the system in significant detail [4,32,33,34,35]. An early report by them on the Na vibrations in Si_28_ showed a nearly *x*-independent “off-center” displacement measured to be approximately 0.4–0.5 Å [32]. Our first-principles calculations correlate well with these experimental results. This is shown in Figure 5, where both the XRD data and our first-principles predictions of the Na displacements in Si_28_ are shown as a function of guest stoichiometry amount *x*. For cases *x* = 12 and *x* = 16, these calculations predict an isotropic displacement that ranges from 0.50 Å for Na_12_Si_136_ to 0.42 Å for Na_16_Si_136_. Another report [34] states elsewhere that an attractive interaction occurs between the neighboring Na guests (situated in adjacent polyhedron cage Si_28_) in the presence of the above discussed anharmonic guest-host potential energy, which causes the “off-center” displacement of the guests to appear.

**Figure 6:**
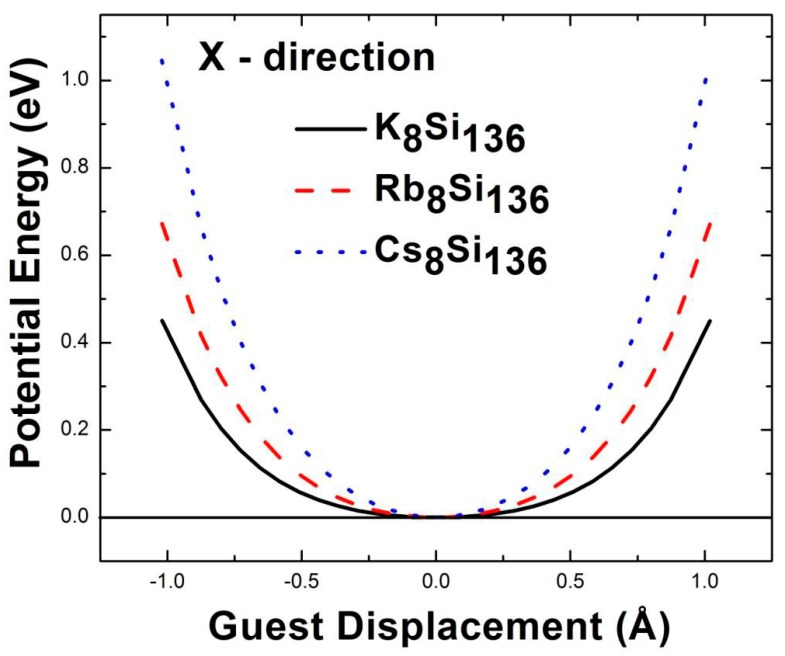
The potential energy curves for alkali atoms K, Rb, and Cs inside the Si framework. X represents the motion direction with respect to the alkali atom.

Utilizing a similar method to study the anharmonic effects associated with the rattling guest atoms K, Rb, and Cs in Si_136_, we show the isotropic guest-framework effective potentials for K_8_Si_136_, Rb_8_Si_136_, and Cs_8_Si_136_ (see Figure 6). The effective potential energy is again symmetric, so only the spring harmonic constant *K_iso_* and the quartic anharmonic coefficient *Ɛ_iso_* need to be considered. That is, the LDA effective potential energy can be fitted to the equation for *V*_eff_, as discussed above. We find that increasing the atomic mass of the rattling guest results in a higher potential energy for a given displacement. After fitting the LDA effective potential to *V*_eff_, the second order and fourth order coefficients for the guest-framework interaction potential (Figure 4 and Figure 6) are obtained; the results are summarized in Table 1.

To examine the anharmonic effects in the guest-host interaction, the self-consistent phonon (SCP) model [36] is used to acquire the anharmonic nature of the phonon rattling modes at nonzero temperature. Accordingly, the temperature-dependent anharmonic rattling frequency (*Ω*(*T*)) at 300 K appearing in Table 1 (denoted by *Ω*_(*T =* 300 K)_) can be quantitatively understood by correlating the thermal displacement *U*_iso_ to the spring constant and quartic anharmonic coefficient arising from potential energy *V*_eff_: *MΩ*^2^(*T*) = *K*_iso_ + *Ɛ*_iso_<*u*^2^(***k***,*j*)>. Here, the average mean square amplitude of the phonon atomic displacement <*u*^2^(***k***,*j*)> (= *U*_iso_) relating to the wavevector (***k***) and phonon branch (*j*) is defined via the Debye model [13], and *M* denotes the atomic mass of the guest rattler.

**Figure 7:**
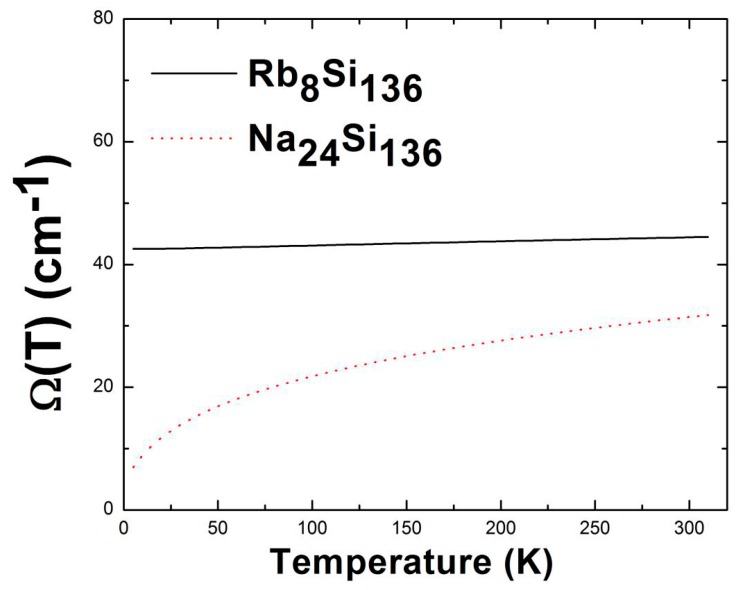
Anharmonic rattling frequency of the guest vibrations in an Si_28_ cage as a function of temperature for the filled binary clathrates Rb_8_Si_136_ and Na_24_Si_136_.

In contrast to the vibration of the Na rattling mode in the Si_28_ polyhedron cage, a very slight variation in temperature-dependent *Ω*(*T*) is observed for the localized Rb impurity in Figure 7. The guest “off-center” displacement has a larger impact on the derived anharmonic rattling frequency and correlated thermal average of <*u*^2^(***k***,*j*)>. Our numerical results describing *Ω*(*T*) in Figure 7 give approximately 42.55 cm^−1^ (≈5.27 meV) for the Rb vibration frequency at *T* = 0, which is approximately 4.2% smaller than that obtained at *T* = 300 K. However, the predicted values of *Ω*(*T*) at 300 K are significantly higher than that at absolute zero for sodium filled clathrate. This strong temperature dependence of *Ω*(*T*) in Na_24_Si_136_ indicates that static disorder effects might impact the isotropic atomic displacement parameters (ADPs). In other words, the average mean square isotropic atomic displacement for Na that resides in the “off-center” system has a boosted value, compared to <*u*^2^(***k***,*j*)> for the “on-center” vibration at the same temperature. Our work predicts that this difference in thermal average <*u*^2^(***k***,*j*)> is nearly close to the square of the “off-center” displacement investigated above (see Figure 5). Intensive discussion on the discrepancy happening to *U*_iso_ is given below.

A previous XRD study by M. Beekman et al. [5] stated that the strong *T*-dependence of <*u*^2^(***k***,*j*)> for Na@Si_28_ in Na*_x_*Si_136_ might be attributable to thermal excitation, which causes the guest to explore the minimum of the “Mexican-hat” shape potential (see Figure 4). That is, the anharmonic and dynamically unstable guest-host interaction makes it possible for the Na rattler to seek a larger vibration volume at elevated temperatures. In other words, the experimentally measured atomic displacement parameters are closely related to the temperature-dependent *Ω*(*T*) at room temperature instead of the DFT-calculated rattling mode *ω*_ph_ when considering the harmonic oscillator model [14], even if there is a large discrepancy between the *Ω*_(*T = 300* K)_ and *ω*_ph_ values.

For the specific case of Na_24_Si_136_, our VASP-calculated result predicts a harmonic rattling frequency that is approximately 15% larger than the anharmonic counterpart *Ω*(*T*) obtained at 300 K. This difference in frequency can lead to differences with respect to the corresponding atomic displacement parameters *U*_iso_. To account for this discrepancy, this effect is interpreted in the picture of the thermal displacement parameter while taking “off-center” displacement due to static disorder into account. Mathematically, we postulate that the relation *U*_iso_(*Ω*_(*T =* 300 K)_) ≈ *U*_iso_(*ω*_ph_) + *d*^2^ can be used to account for the existence of the strong ADPs observed by means of XRD, INS, or temperature-dependent heat capacity (*C*_p_) measurements [5,31,37]. Numerical values of *Ω*_(*T =* 300 K)_ computed by the self-consistent phonon model and *ω*_ph_ given by the VASP code can be plugged into the classical equation *U*_iso_(*ω*) = *k*_B_*T*/*Mω*^2^ within a high-temperature approximation [38]. For the case of Na_4_Si_136_, the calculation leads to the corresponding ADPs, which give approximately 0.497 Å^2^ and 0.122 Å^2^ for *U*_iso_(*Ω*_(*T =* 300K)_) and *U*_iso_(*ω*_ph_) in Na_4_Si_136_, respectively. Therefore, the calculated Na “off-center” displacement *d* in Na_4_Si_136_ is approximately equal to 0.61 Å, which is reasonably comparable to the experimental counterpart (~0.52 Å) found in Figure 4. Similarly, our calculated *d* value by virtue of the suggested model for Na_24_Si_136_ is 0.28 Å, which is in good agreement with the results estimated by Rietveld analysis in Ref. [32]. Moreover, the DFT-calculated “off-center” displacement of Na in Na_24_Si_136_ (see Figure 5) is roughly equal to 0.36 Å, which is slightly higher than our numerically determined *d* parameter.

## 4. Conclusions

We have employed *ab initio* DFT to systematically investigate the electronic, vibrational, and anharmonic properties of the binary clathrate system A_x_Si_136_. One of the main electronic characteristics is that the electronic density of states remains nearly unchanged as the Na composition *x* changes from 8 to 12 to 16 in Na*_x_*Si_136_. Furthermore, the determined Fermi energy level *E*_F_ is shifted to a deep portion of the conduction band in the same range (8 ≤ *x* ≤ 16), while a similar effect characterized by a “double-peak” along with a dip near the Fermi energy in the EDOS calculation is observed in Na_8_Si_136_ and Na_16_S_i136_, in analogy to the (Na,K)_16_Rb_8_Si_136_ case reported previously. Using an effective potential-energy curve to explore the Na vibration in the Si_28_ cage, we point out the existence of a dynamically unstable state in the “double-well” structure. This is also indicative of an “off-center” displacement for guests subjected to elevated temperatures. In addition, the guest vibrations are nearly isotropic in the same stoichiometry Na*_x_*Si_136_ (0 < *x* ≤ 24), independent of the composition *x.* Furthermore, the DFT-determined “off-center” displacement for the Na vibration in the Si_28_ cages is nearly independent of guest content, in fair agreement with the XRD data. It is noted that the quartic anharmonicity effect induced by the guest-framework interaction becomes comparably weakened when the ratio of the lowest even anharmonic coefficient to the spring constant decreases in the A_8_Si_136_ system. Making use of the SCP model, the determined temperature-dependent rattling frequency *Ω*(*T*) of Na in Na_24_Si_136_ implies strong static disorder effects associated with the average mean square isotropic atomic displacement *U*_iso_. This is manifested by the fact that the values of *Ω*(*T*) for Na at 300 K are approximately six times larger than the value at absolute zero. To account for the experimental overestimation of *U*_iso_ at room temperature, we suggest that the existence of strong ADPs observed by means of XRD, INS, or temperature-dependent heat capacity (*C*_p_) measurements in apparently anharmonic clathrate Na_24_Si_136_ is offset by the square of the Na “off-center” displacement in the Si_28_ cage. Considering the ability of tuning the alkaline guest (A) species and its composition *x* in such a “cage-structured” configuration of A_x_Si_136_, theoretical investigation on the corresponding rattling behavior, as well as guest “off-center” displacement, may contribute to boosting the search for novel thermoelectric materials. A good performance of TE material is manifested and parameterized by high electric conductivity and low thermal conductivity, according to “Phonon Glass Electron Crystal” criteria [28]. Therefore, fabrication of this class of clathrates may be used in TE technology involving the conversion of heat to electricity, and in optoelectronics technology, including the conversion of light to electricity. Accordingly, recent work on alloyed Si-Ge type II clathrate [39] presents many promising optoelectronic applications, which are pertinent to single junction PV cells with optimized band gaps and Si-Ge multijunction cells.

## Figures and Tables

**Table 1:** Fitting parameters of the guest-host interaction energy potentials for A_8_Si_136_ (A = Na, K, Rb, Cs) and Na_24_Si_136_.

Material	*K*_iso_ (eV/Å^2^)	*ε*_iso_ (eV/Å^4^)	*ω*_ph_ (cm^−1^)	*Ω*_(*T* = 300 K)_ (cm^−1^)	(*K*_iso_/*M*)^1/2^ (cm^−1^)
Na_8_Si_136_	−0.143	0.456	33.59	26.31	-
Rb_8_Si_136_	0.565	1.389	37.59	44.41	42.29
Cs_8_Si_136_	1.035	1.857	43.61	46.90	45.90
K_8_Si_136_	0.292	1.095	47.12	50.54	44.95
Na_24_Si_136_	−0.061	0.467	36.27	31.43	-
